# Sequencing and Variant Detection of Eight Abundant Plant-Infecting Tobamoviruses across Southern California Wastewater

**DOI:** 10.1128/spectrum.03050-22

**Published:** 2022-11-14

**Authors:** Jason A. Rothman, Katrine L. Whiteson

**Affiliations:** a Department of Molecular Biology and Biochemistry, University of California, Irvine, Irvine, California, USA; USDA–San Joaquin Valley Agricultural Sciences Center

**Keywords:** metatranscriptomics, plus-strand RNA virus, tobamovirus, wastewater, wastewater-based epidemiology

## Abstract

Tobamoviruses are agriculturally relevant viruses that cause crop losses and have infected plants in many regions of the world. These viruses are frequently found in municipal wastewater, likely coming from human diet and industrial waste across wastewater catchment areas. As part of a large wastewater-based epidemiology study across Southern California, we analyzed RNA sequence data from 275 influent wastewater samples obtained from eight wastewater treatment plants with a catchment area of approximately 16 million people from July 2020 to August 2021. We assembled 1,083 high-quality genomes, enumerated viral sequencing reads, and detected thousands of single nucleotide variants from eight common tobamoviruses: bell pepper mottle virus, cucumber green mottle mosaic virus, pepper mild mottle virus, tobacco mild green mosaic virus, tomato brown rugose fruit virus, tomato mosaic virus, tomato mottle mosaic virus, and tropical soda apple mosaic virus. We show that single nucleotide variants had amino acid-altering consequences along with synonymous mutations, which represents potential evolution with functional consequences in genomes of these viruses. Our study shows the importance of wastewater sequencing to monitor the genomic diversity of these plant-infecting viruses, and we suggest that our data could be used to continue tracking the genomic variability of such pathogens.

**IMPORTANCE** Diseases caused by viruses in the genus Tobamovirus cause crop losses around the world. As with other viruses, mutation occurring in the virus’s genomes can have functional consequences and may alter viral infectivity. Many of these plant-infecting viruses have been found in wastewater, likely coming from human consumption of infected plants and produce. By sequencing RNA extracted from influent wastewater obtained from eight wastewater treatment plants in Southern California, we assembled high-quality viral genomes and detected thousands of single nucleotide variants from eight tobamoviruses. Our study shows that Tobamovirus genomes vary at many positions, which may have important consequences when designing assays for the detection of these viruses by agricultural or environmental scientists.

## OBSERVATION

Wastewater represents a matrix of microorganisms, human waste, and water inflow across a sewage catchment area (1). As part of the microorganismal fraction of wastewater, there are often high abundances of plant-infecting positive-sense single-stranded RNA viruses of the genus *Tobamovirus*, which represents important plant pathogens causing substantial crop losses to the global agricultural industry (2 to 6). These viruses are required to be tested for before importation by the United States Department of Agriculture, as infections have been reported both in the United States and internationally (7 to 10). Tobamoviruses are widespread and may be deposited into wastewater through agricultural runoff and human diet, where they can resist degradation even through wastewater and drinking water treatment (2). They are often the most abundant RNA viruses in human feces and wastewater samples (2), even going back to the first human fecal RNA virome sequenced (11). For example, pepper mild mottle virus is ubiquitous in wastewater and can remain infectious in effluent even after wastewater treatment (12). As part of ongoing efforts and advances in wastewater-based epidemiology (WBE), it is critical to monitor wastewater for the presence of tobamoviruses and their potential to infect new hosts or evade plant immunity (13). Also, as many tobamoviruses may serve as water quality indicators and are impactful diseases to agriculture, studies should be conducted to understand the genomics of these viruses (2, 14).

As part of a large WBE effort across Southern California, we used metatranscriptomic sequencing to investigate the genomics and single nucleotide variants (SNVs) of eight tobamoviruses sourced from 275 samples across eight wastewater treatment plants from July 2020 to August 2021 (3, 15). These viruses were bell pepper mottle virus (BPeMV), cucumber green mottle mosaic virus (CGMMV), pepper mild mottle virus (PMMoV), tobacco mild green mosaic virus (TMGMV), tomato brown rugose fruit virus (ToBRFV), tomato mosaic virus (ToMV), tomato mottle mosaic virus (ToMMV), and tropical soda apple mosaic virus (TSAMV). Through our study, we investigated several lines of inquiry. Can we assemble high-quality *Tobamovirus* genomes from wastewater samples? Do we obtain acceptable sequencing coverage across viral genomes derived from wastewater? Can we identify SNVs across tobamoviruses in Southern California’s wastewater?

**FIG 1:**
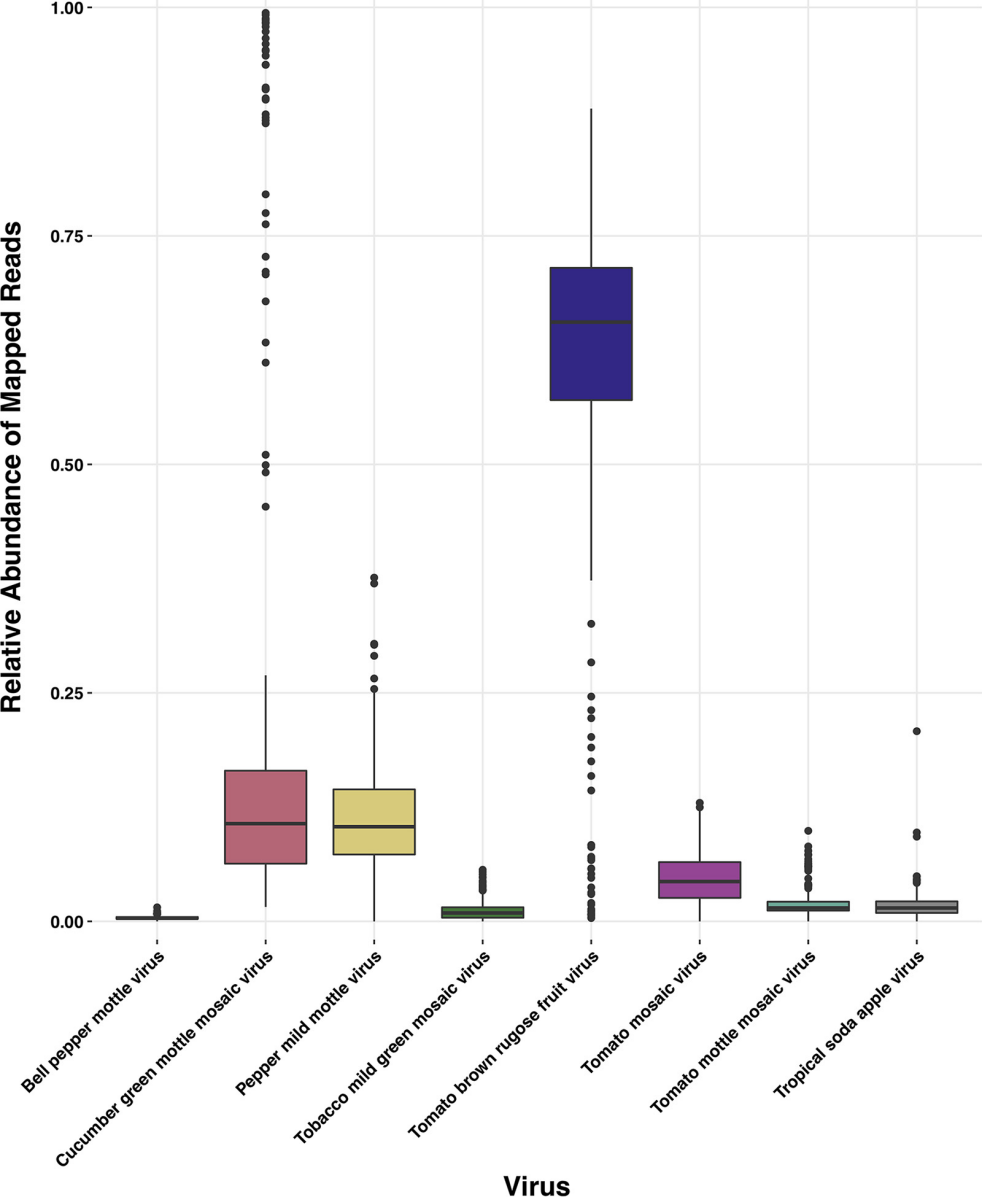
Boxplots of the average relative abundances of mapped reads (within this study only) of each *Tobamovirus* across all samples. Lines within each box represents the median relative abundance, whiskers are 1.5× the interquartile range (IQR), and dots are values >1.5 IQR.

**Results.** We aligned 156,825,269 quality-filtered, deduplicated, matching paired-end reads (313,650,538 individual reads) across 275 samples from eight water treatment plants (average = 570,274 paired-end reads, range = 44 to 8,933,433). Of the paired-end reads that mapped to the eight tobamoviruses, 0.34% were bell pepper mottle virus (BPeMV), 12.90% were cucumber green mottle mosaic virus (CGMMV), 11.90% were pepper mild mottle virus (PMMoV), 1.31% were tobacco mild green mosaic virus (TMGMV), 64.06% were tomato brown rugose fruit virus (ToBRFV), 5.6% were tomato mosaic virus (ToMV), 1.87% were tomato mottle mosaic virus (ToMMV), and 2.02% were tropical soda apple mosaic virus (TSAMV) (Fig. 1; Fig. S1 in the supplemental material).

**FIG 2:**
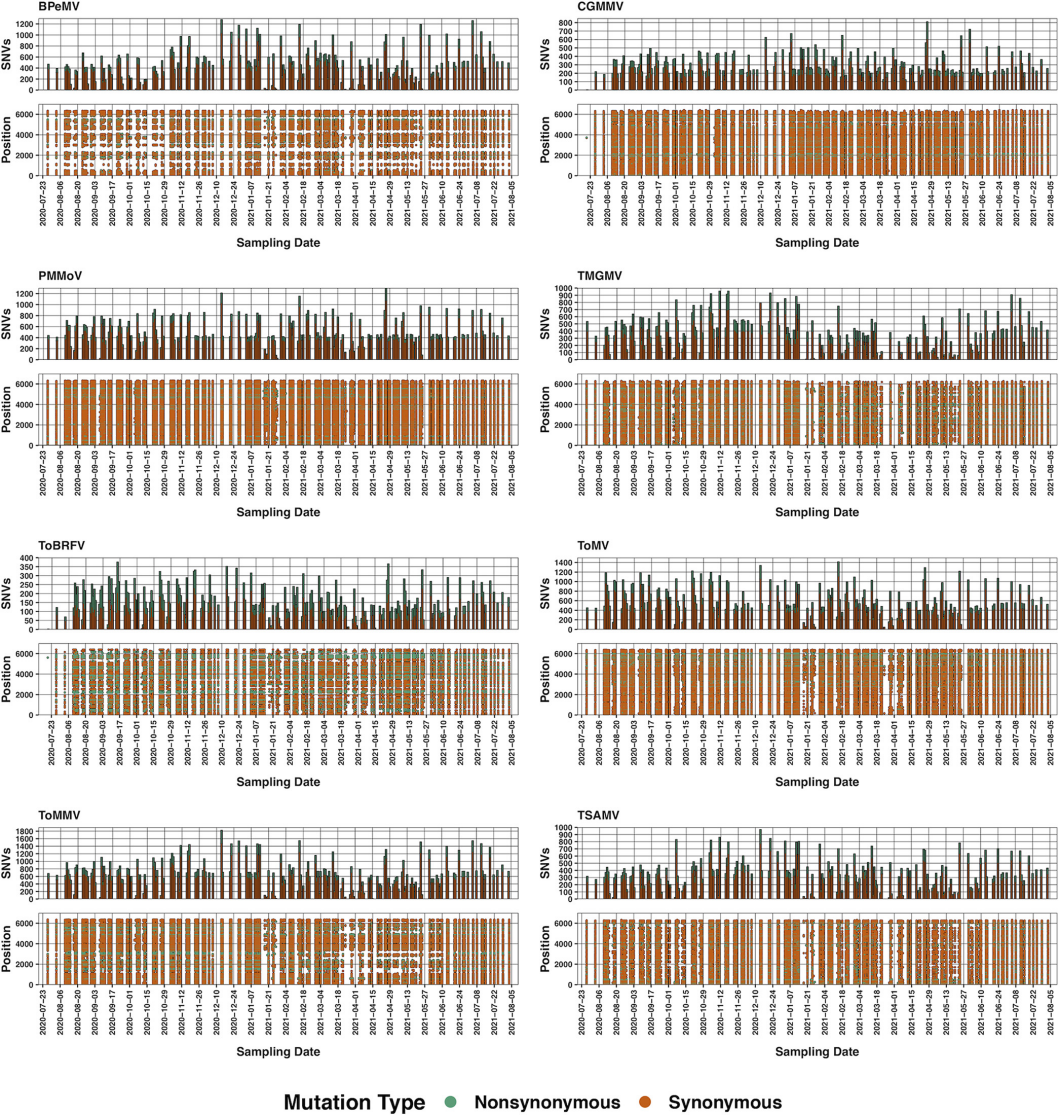
Genomic position and number of single nucleotide variants (SNVs) for bell pepper mottle virus (BPeMV), cucumber green mottle mosaic virus (CGMMV), pepper mild mottle virus (PMMoV), tobacco mild green mosaic virus (TMGMV), tomato brown rugose fruit virus (ToBRFV), tomato mosaic virus (ToMV), tomato mottle mosaic virus (ToMMV), and tropical soda apple mosaic virus (TSAMV) at each sampling date. Plot coloration denotes mutational consequence of the SNV.

For each virus, we report the total number, average, and range of mapped paired-end reads, the average sequencing depth and overall genomic coverage, the number of high-quality assembled genomes, the minimum DIAMOND alignment percentage, and the number of single nucleotide variants (SNVs) along with the SNVs’ mutational consequence (synonymous or nonsynonymous) in Table 1. We also plotted the average read depth per nucleotide (Fig. S2) and the genomic position and date of each SNV detected along with its mutational consequence for each virus (Fig. 2). Lastly, we provide the relevant iVAR output for each sample and SNV along with the sequences of all high-quality viral genomes on Dryad (doi.org/10.7280/D1S69X) (16).

**TABLE 1:** Sequencing results for each virus across all viruses

Virus	Mapped paired-end reads	Avg/range mapped reads per sample	Avg sequencing depth per nucleotide and breadth	No. of high-quality genomes	Alignment percentage of genomes to reference strain	SNVs^*a*^
BPeMV	526,713	1,915 (0 to 26,075)	84 × 99.5%	0	NA	963: dN = 241, dS = 722
CGMMV	20,222,039	73,535 (6 to 1,248,760)	2,347 ×100%	144	>99.5%	1,384: dN = 397, dS = 987
PMMoV	18,663,282	67,866 (0 to 1,028,539)	2,130 ×100%	90	>99.3%	1,306: dN = 381, dS = 925
TMGMV	2,057,301	7,481 (0 to 116,449)	240 × 100%	141	>97.6%	1,557: dN = 509, dS = 1,048
ToBRFV	100,455,804	365,294 (1 to 6,727,467)	11,854 × 100%	250	>99.9%	1,075: dN = 452, dS = 623
ToMV	8,788,061	31,957 (0 to 513,129)	1,162 × 99.9%	183	>99.6%	1,540: dN = 429, dS = 1,111
ToMMV	2,939,466	10,689 (0 to 120,789)	487 × 99.9%	79	>98.6%	1,309: dN = 372, dS = 937
TSAMV	3,172,603	11537 (0 to 200383)	360 × 100%	196	>99.4%	1,531: dN = 486, dS = 1045

a SNVs, single nucleotide variants.

**Discussion.** Wastewater-based epidemiology (WBE) has been used to characterize pathogen abundances and genomics for a variety of diseases and is often employed to detect antibiotic resistance or diseases relevant to public health (5). We applied similar molecular and bioinformatic methods to eight agriculturally relevant tobamoviruses sequenced from influent wastewater, representing a sewer shed of approximately 16 million Southern Californians across eight wastewater treatment plants (3, 15). These tobamoviruses were abundant and widespread throughout our wastewater samples, comprising 8 of the top 10 viruses in our data set (15). PMMoV may be the best known and is often regarded as the most abundant virus in fecal and wastewater samples (11, 14); however, we were surprised to find that ToBRFV was much more abundant, mirroring the results of a recent study from Maryland (5). Likely due to their near-ubiquity, we obtained very deep and broad sequencing coverage across their genomes. Our samples yielded thousands of SNVs per virus, and we assembled over 70 individual high-quality composite genomes for each viral species except BePMV, supporting studies that have suggested WBE is useful in characterizing the genomic landscape of pathogens (3, 5, 6, 17). We also recognize that our assembled genomes were from composite samples and are likely not true whole genomes, but rather represent a consensus of the individual genomes. Interestingly, most of the SNVs identified were synonymous mutations, although there were thousands of putative nonsynonymous mutations that may have consequences in host infectivity or immune escape.

As tobamoviruses are being developed for use as water-quality indicators, it is important to have a broad pool of wastewater-sourced genomes, sequences, and SNVs so that proper tests can be developed that reflect the diversity of each virus (2, 14). For example, *Tobamovirus* testing involves careful selection of specific, validated RT-qPCR primers, which may lose specificity as viral mutations arise, making pathogen detection unreliable without adjusting for new variants (18). Likewise, to combat outbreaks of tobamoviruses, or the evolution of novel viruses, deep sequence resources should be provided to the scientific and agricultural communities (13). To the best of our knowledge, our study is the first to report such a wide diversity of *Tobamovirus* SNVs from wastewater, and we suggest that future research be conducted using WBE for other agriculturally relevant diseases. Furthermore, as water reuse is becoming widespread, studies should investigate the ability of wastewater treatment plants to inactivate tobamoviruses to prevent accidental infection through irrigation and to indicate expected decreases in viral load for public health (14).

**Materials and methods.** We obtained raw sequencing data as FASTQ files from the NCBI Sequence Read Archive under BioProject PRJNA729801, and we refer to Rothman et al., 2021 (3) and Rothman et al., 2022 (15) for all sampling, RNA extraction, and sequencing methods. We used BBTools (19) “bbduk” to remove sequencing adapters, primers, and low-quality bases from the reads and BBTools “dedupe” to remove optical duplicates, and removed human genome reads (hg38) with Bowtie2 (20). We then used Bowtie2 to align the reads to the reference strains (downloaded from NCBI) for each *Tobamovirus*: BPeMV (NC_009642.1), CGMMV (NC_001801.1), PMMoV (NC_003630.1), TMGMV (NC_001556.1), ToBRFV (NC_028478.1), ToMV (NC_002692.1), ToMMV (NC_022230.1), and TSAMV (NC_030229.1) and calculated the relative abundance by dividing the number of reads that mapped to each independent virus with reads that mapped to all eight viruses with SAMtools (21).

We used SAMtools (21) to assess sequencing depth and breadth of genomic coverage on the BAM files. We then used iVar (22) to identify single nucleotide variants (SNVs) for each virus in each sample separately and plotted the SNVs and genome depth/coverage in R (23) using “ggplot2” (24) and “patchwork” (25). We assembled contigs within each sample with MEGAHIT (26) and assessed contig assembly quality with checkV (27), using a cutoff of >90% completeness and 0% contamination to characterize them as “high-quality genomes.” We used DIAMOND (28) to classify the “high-quality genomes” and plotted summary statistics about each sample and virus with “ggplot2.”

**Data availability.** Data used in this study are available on the NCBI Sequence Read Archive (SRA) under BioProject accession number PRJNA729801 and on the Dryad Digital Repository (doi.org/10.7280/D1S69X) (16). We report the individual SRA and BioSample accession numbers and the study each sample’s’ data were obtained from in Supplemental File 1.
