# Supplementary material for: Sequencing and Variant Detection of Eight Abundant Plant-Infecting Tobamoviruses across Southern California Wastewater
Source: Microbiol Spectr. 2022 Nov 14;10(6):e03050-22. doi: 10.1128/spectrum.03050-22 (PMC9769696; doi:10.1128/spectrum.03050-22)
Supplement: Supplemental file 2 — Supplemental material. Download spectrum.03050-22-s0001.pdf, PDF file, 0.7 MB [file spectrum.03050-22-s0001.pdf]

Supplemental information for:

Sequencing and variant detection of eight abundant plant-infecting tobamoviruses across Southern California wastewater.

Jason A. Rothman[a#] and Katrine L. Whiteson[a]

[a] Department of Molecular Biology and Biochemistry, University of California, Irvine, Irvine, CA, USA.

# Corresponding author: Jason A. Rothman, University of California, Irvine, Irvine, CA, 92697, (949) 824-3509, rothmanj@uci.edu.

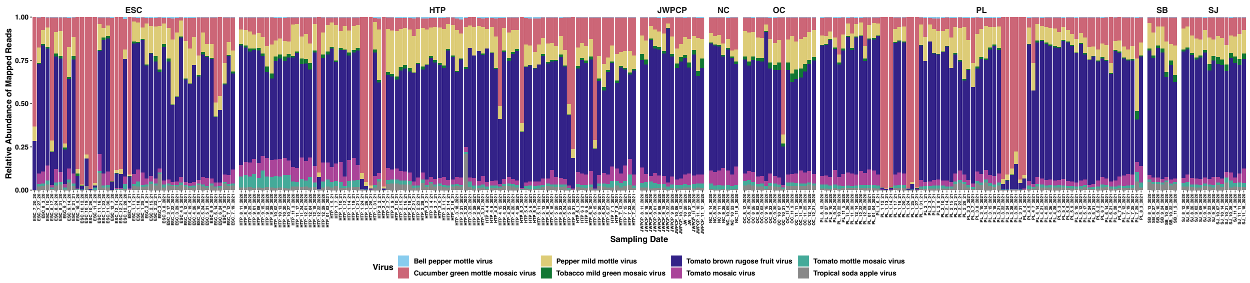

Figure S1: Stacked bar plot of the relative abundances of mapped paired-end reads for each *Tobamovirus* in individual samples within this study, faceted by water treatment plant. Colors denote virus identity.

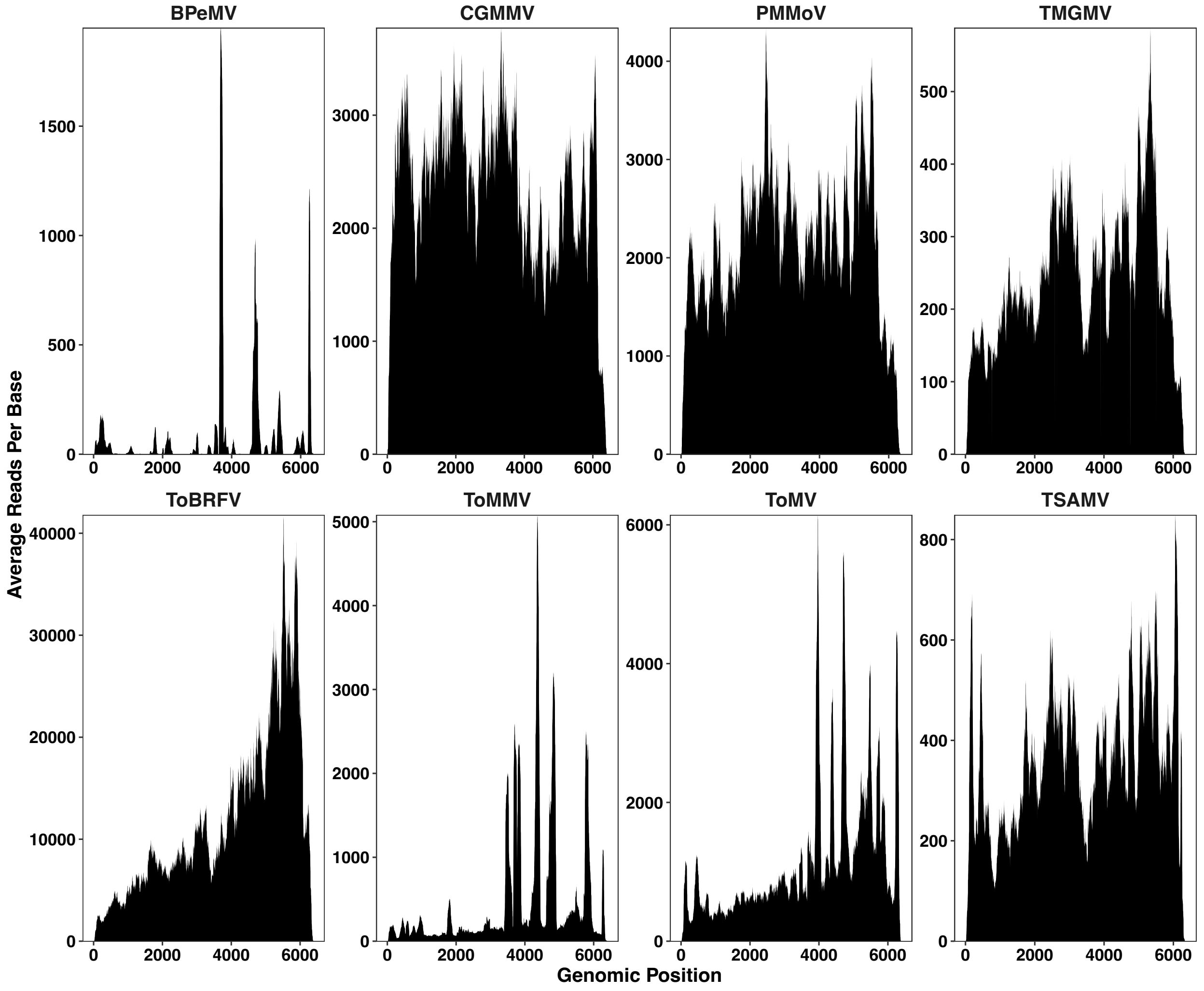

Figure S2: Area plot of the average paired-end mapped reads per base across all samples for each *Tobamovirus* faceted by virus. X-axes represent the genomic nucleotide position within each virus.

Supplemental file SF1: Sample IDs, Sequence Read Archive and BioSample accession numbers, and the study that each sample was sequenced in represented by either "Rothman et al 2021" (1) or "Rothman et al 2022" (2).

Supplemental references:

1. Rothman JA, Loveless TB, Kapcia J 3rd, Adams ED, Steele JA, Zimmer-Faust AG, Langlois K, Wanless D, Griffith M, Mao L, Chokry J, Griffith JF, Whiteson KL. 2021. RNA viromics of Southern California wastewater and detection of SARS-CoV-2 single-nucleotide variants. Appl Environ Microbiol 87:e0144821.

2. Rothman JA, Saghir A, Chung S-A, Boyajian N, Dinh T, Kim J, Oval J, Sharavanan V, York C, Zimmer-Faust AG, Langlois K, Steele JA, Griffith JF, Whiteson KL. 2022. Longitudinal metatranscriptomic sequencing of Southern California wastewater representing 16 million people from August 2020-21 reveals widespread transcription of antibiotic resistance genes. bioRxiv. doi: https://doi.org/10.1101/2022.08.02.502560
